# Realistic Indoor Radio Propagation for Sub-GHz Communication

**DOI:** 10.3390/s18061788

**Published:** 2018-06-01

**Authors:** Ben Bellekens, Rudi Penne, Maarten Weyn

**Affiliations:** 1 IDLab, Faculty of Applied Engineering, University of Antwerp-imec, Groenenborgerlaan 171, 2000 Antwerp, Belgium; maarten.weyn@uantwerpen.be; 2 Op3Mech, Faculty of Mathematics and Applied Engineering, University of Antwerp, Groenenborgerlaan 171, 2000 Antwerp, Belgium; rudi.penne@uantwerpen.be

**Keywords:** propagation loss model, indoor, ray launching, realistic, validation, sub-GHz, SLAM, Quadtree, LPWAN

## Abstract

This research article proposes a novel ray-launching propagation loss model that is able to use an environment model that contains the real geometry. This environment model is made by applying a Simultaneous Localization and Mapping (SLAM) algorithm. As a solution to the rising demands of Internet of Things applications for indoor environments, this deterministic radio propagation loss model is able to simulate an accurate coverage map that can be used for localization applications or network optimizations. Since this propagation loss model uses a 2D environment model that was captured by a moving robot, an automated validation model is developed so that a wireless sensor network can be used for validating the propagation loss model. We validated the propagation loss model by evaluated two environment models towards the lowest Root Mean Square Error (RMSE), the Mean Absolute Error (MAE), and the Mean Error (ME). Furthermore, the correlation between the number of rays and the RMSE is analyzed and the correlation between the number of reflections versus the RMSE is also analyzed. Finally, the performance of the radio propagation loss model is analyzed.

## 1. Introduction

Nowadays, the need for a good and stable wireless connectivity at any indoor location and any time has grown to a necessity in our current daily lives due to the growing demands of Internet of Things (IoT) applications. Because of this, the need for an algorithm that is able to indicate the places where an IoT sensor has no or limited reception has grown. Such an algorithm or propagation loss model simulates the received signal strength (RSS) or more fundamental, the electrical field according to an environment model and the principals of electromagnetic wave propagation. Generally, radio propagation loss models are subdivided into two types [1,2,3]. First, deterministic algorithms require a full environment model that has all objects included with their specific electrical material parameters so that the influence of the wave propagation phenomena can be included in the resulted signal strength computation [4,5,6,7]. Second, statistical algorithms do not take the different phenomena into account, which makes it less complex, fast to compute, and less suitable for validating an accurate and precise indoor localization algorithm because of the large impact of multipath [1,8,9,10].

This paper presents a novel ray-launching propagation loss model that is categorized as a deterministic model. This propagation loss model uses an environment model that was built with a robot. Moreover, this environment model is built from the combination of a laser scanner and the relative motions of the robot. In order to combine both data streams, a simultaneous localization and mapping (SLAM) algorithm is applied [11,12]. After this, the individual points of the obtained occupancy map are inserted in a volumetric space representation called Quadtree. This makes it possible to describe an environment according to a specific resolution. Next, the implementation of the propagation loss model is based on four parts. The first part targets the extraction of line segments from points and the segmentation of walls and objects by applying a region growing connected component labeling algorithm on the Quadtree. Second, a transmitter configuration is built, which specifies the amount of rays, operating frequency, the antenna pattern, and the number of reflections. Third, the ray calibration launches every ray in the environment model according to a specified antenna pattern. This process generates a list for every ray that exists of all visited cells that contain the total distance and the reflection coefficients. Finally, the electrical field at every visited cell for each ray is being computed in the last part. As a result, a coverage map can be created by computing the sum of all electrical fields at every cell that was visited.

The opportunity of mapping a real indoor environment related to the current location and vice versa leads to the following advantage. Using a robotic algorithm for simulating the signal strength has the ability of estimating the locations relative to the first location. This makes it possible to compute all locations where signal strength measurements were taken and will result in an automated validation solution that empowers the usability of radio propagation algorithms [7]. Such an automated solution can be used in different application domains such as indoor/outdoor localization systems, telecommunications systems, and wireless network systems to optimize the results based on realistic signal strengths simulations [3,13,14].

To validate and quantify our ray-launching propagation loss model so that an optimal simulation configuration can be found, two different indoor office environments are used. Both environments were equipped with several transmitters that were operating at 433.0 MHz and were able to broadcast a packet according to the sub-GHz mid-range DASH7-standard [15]. On the other hand, the robot that was used to map the environment was also equipped with a receiver that was able to receive a DASH7-packet. By using the automated validation approach, a large number of individual links can be analyzed in order to get a robust evaluation of the propagation model. As a result, the accuracy that is defined as the root mean square error (RMSE), the mean absolute error (MAE), and the mean error (ME) will be evaluated regarding the resolution of the environment, the number of rays, and the maximum level of reflections that is allowed. Next, the performance of the propagation loss model is analyzed related to the number of processing units and the resolution of the environment map. The aim of this research article is to indicate the optimal simulation configuration where the accuracy and the precision are minimal.

This paper is structured as follows: Section 2 explains the current scope of the related literature. Section 3 covers the methods that model the environment with a moving robot. Next, Section 4 describes the different steps of the ray-launching propagation loss model by using a generic environment model. Section 5 explains the automatic validation system, which is used in Section 6 to evaluate both environments towards a robust accuracy and precision. Finally, the conclusions are proposed in Section 7.

## 2. Related Literature

Within the scope of this article, we will focus on the implementation and validation of a novel deterministic propagation loss model. Deterministic radio propagation loss models solve the original Maxwell’s equations according to the integral or differential solution. In the literature, two types of models are proposed and are also called site-specific models:

1. Ray-based algorithms that make it possible to trace a set of rays in the modeled environment until a specific location. Every ray that is either traced or launched from a transmitter location and is able to reflect, refract, or diffract in the environment by using the Geometric Optics (GO) theory and the Uniform Theory of Diffraction (UTD). Ray-based methods enable the visibility of every path from transmitter to receiver, which gives researchers a better understanding of the underlying phenomena within the frequency spectrum. On one hand, the literature states an image-based solution, which traces all ray paths from a receiver location towards a transmitter location [10,16,17]. On the other hand, a ray-launching solution makes it possible to launch many different rays according to a specific antenna radiation pattern. Since both solutions are providing a good accuracy, they are widely being applied for either outdoor and indoor coverage simulations [13].
2. Finite Difference Time Domain (FDTD) and Method of Moments (MoM) models are solutions that solve the Maxwell’s equations in both time and frequency domains [18,19]. All of these deterministic propagation loss model solutions use a grid-based environment model, which can define a 2D or a 3D environment model, to compute at every cell the electrical field relative to the time according to the differential or integral method. The different wave propagation phenomena make such an algorithm very complex on one side, while these algorithms obtain the optimal accuracy and precision for the given environment model relative to the resolution of the obtained environment [6].

Furthermore, a more detailed explanation is discussed about ray-based methods to provide a fundamental background of existing solutions. Ray-based methods can be divided into two categories: ray-tracing and ray-launching methods [5,6]. Ray-tracing methods determines all rays between the receiver and the transmitter by applying the image based technique. This technique is able to find all reflections starting from the source (receiver) until the transmitter in a recursive way by mirroring the source point with the reflecting plane. Second, the mirrored source point can then be connected with the transmitter location in order to compute the reflection intersection point. This principle can further be extended in order to find multiple reflections so that all paths can be found. Since this process is very time-consuming, this technique is mostly being used for simulating point-to-point connections. Alternatively, ray-launching methods calculate all paths starting from the transmitters. This technique launches *n* numbers of rays in different directions, which can be modeled according to a specific antenna radiation pattern. Ray-launching propagation loss models are more efficient than ray-tracing methods, which makes them suitable for coverage predictions. Two examples are further explained. First, Klepal [19] designed a semi-deterministic propagation loss model that is called the Motif model. This model defines an environment as a regular grid with m×n cells. In addition to this, each cell that is part of an object or environment segment is called a Motif, which is represented by probabilistic motif parameters. These motif parameters include the probability of absorption of a ray and the probability radiation pattern when a ray intersects the Motif cell. Furthermore, the Motif model implemented a Monte Carlo principle that enables splitting up a ray in many sub-rays when it intersects a Motif element. These sub-rays will be launched according to the overall probabilistic radiation pattern. Second, Lai et al. [20] implemented the indoor Intelligent Ray Launching Algorithm (IRLA) model. This model is based on a discrete ray-launching principle that launches *n* number of rays in the environment model that is modeled as a regular grid according to a specific antenna radiation pattern. Next, three main components are applied to simulate the received signal strength: the Horizontal Reflection Diffraction (HRD), the Line-of-Sight (LoS), and the Vertical Diffraction (VRD). The Indoor IRLA model applies a calibration process to optimize the material parameters according to measurements, which enhances the accuracy of the validation. Next, both ray-based propagation loss models do not include phase difference, which results in the fact that constructive and destructive wave propagation concept are not included.

## 3. Environment Modeling

This section explains the process of mapping an environment by using a robot so that it can be used in a propagation loss model. This map represents the occupied space as well as the free space and will be used in Section 4 to simulate the signal strength. Together with the occupancy grid-map, the traveled path is created by applying a simultaneous localization and mapping algorithm (SLAM). As a result of this, all points that represent the environment are inserted in a Quadtree, which is a tree-based data structure.

### 3.1. SLAM

For many years, researchers have come up with solutions to the SLAM problem. This problem occurs when a robot is placed in an unknown environment. Without a map or information about its own location, the robot has to be able to build a map of the environment while determining its own position at the same time [21,22,23,24]. Applying such an algorithm is by far one of the most important steps towards autonomous driving cars and realistic radio propagation simulation.

Subsequently, SLAM is usually built by the combination of at least two parts: one, a scan or sensor measurement of the area that is taken with a laser or a depth-sense camera; two, the relative motion of each movement of the robot that is measured by two wheel encoders. When both data streams are interpreted as multivariate Gaussian distributions, they can be combined in a probabilistic fashion by using a Kalman filter so that the belief of the location will increase while the map will increase. On the other hand, all kinds of artefacts and mismatches can occur in this system, which makes this topic a challenge for researchers. Within this research article, a SLAM algorithm that uses a Rao–Blackwellized particle filter is being used. This algorithm is mainly optimized for long-range laser scanners and implements a new technique to prevent particle depletion, which results in a higher pose belief [12].

### 3.2. Robot

**Figure 1:**
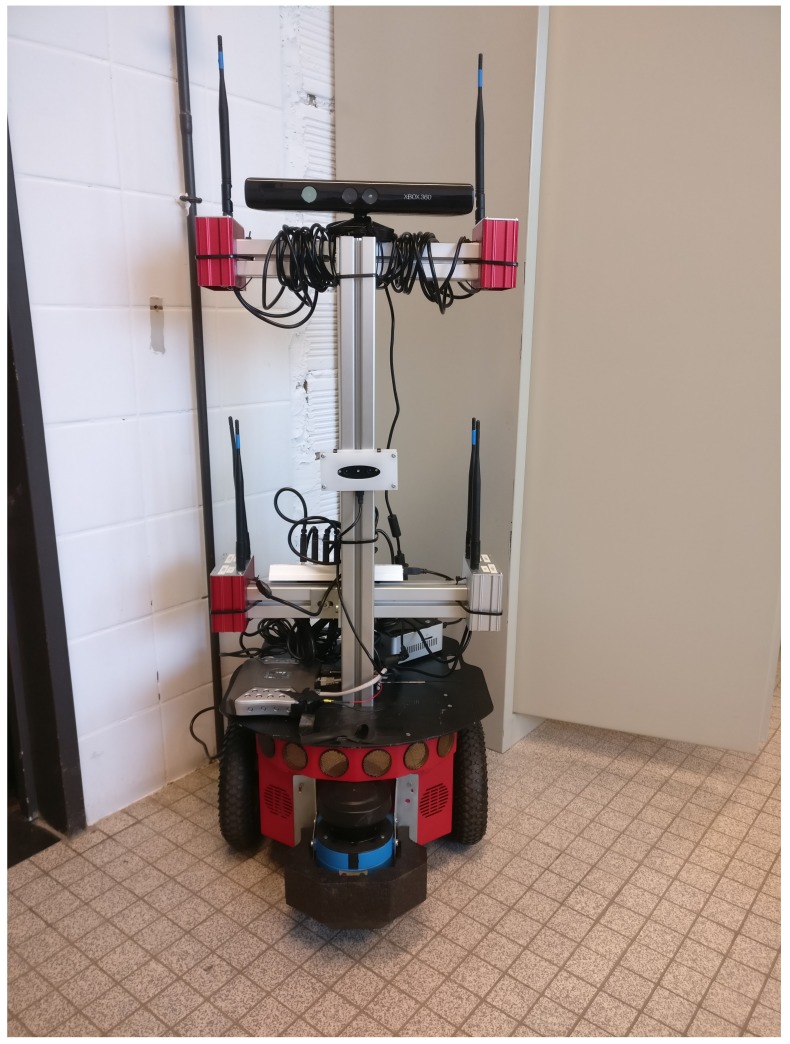
Picture of the Pioneer 3DX robot that was used for validating the propagation model.

The robot that was used to map the test environments is the compact differential-drive Pioneer 3-DX. This robot is worldwide being used for academic research about environment mapping, localization, autonomous driving, etc. It contains two wheel encoders that exist of 500-ticks, which result in a high accuracy odometry reading. Furthermore, it is powered by three lead-acid batteries that each deliver 7.2 Ah. Next, an aluminum frame is mounted on top of the robot that contains four radio receivers, a stereo camera, and a depth-sense camera. In addition to this, a LIDAR is mounted in front of the robot together with a high performance computer and an access point to monitor the robot remotely as can be seen in Figure 1.

This computer operates the robot via the Robotic Operating System (ROS), which provides all kinds of hardware drivers, libraries to map an environment, and tools to control the robot on one hand and read all the sensor measurements on the other hand. Furthermore, ROS is a centralized time-based framework that can process different applications or nodes according to a specific geometrical transformation that is related to the time that the measurement was processed. This delivers an important advantage to validate the propagation loss model because all radio measurements can be mapped onto the geometrical transformation of the mapped environment and thus each transmitter location in the environment relative to the initial robot location is known. The indicated advantage reduces the installation time to validate the propagation model because only the locations of the fixed nodes in the environment need to be measured relative to the first robot location.

### 3.3. Quadtree

**Figure 2:**
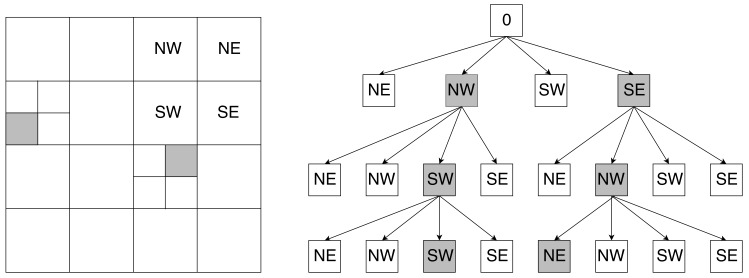
This figure illustrates a Quadtree hierarchical data structure where the gray cells indicate the route toward the two gray indicated occupied leaves.

A Quadtree is a hierarchical data structure that can contain 2D spatial information like a point or a line segment [25,26]. The recursive decomposition of a Quadtree divides every node, which represents a square space, in four equal leaves as can be seen in Figure 2.

In this figure, every leaf is indexed according to northeast (NE), northwest (NW), southwest (SW), and southeast (SE). This makes it possible to implement an efficient search algorithm on top of the data structure. Moon et al. [27] described a survey of different search patterns or space-filling techniques, of which the Hilbert curve achieves the best results. Such a technique tries to find the most efficient pattern for traversing and indexing a spatial data structure. Furthermore, they provided algebraic closed-form solutions to expect the number of cluster for a given query region.

**Figure 3:**
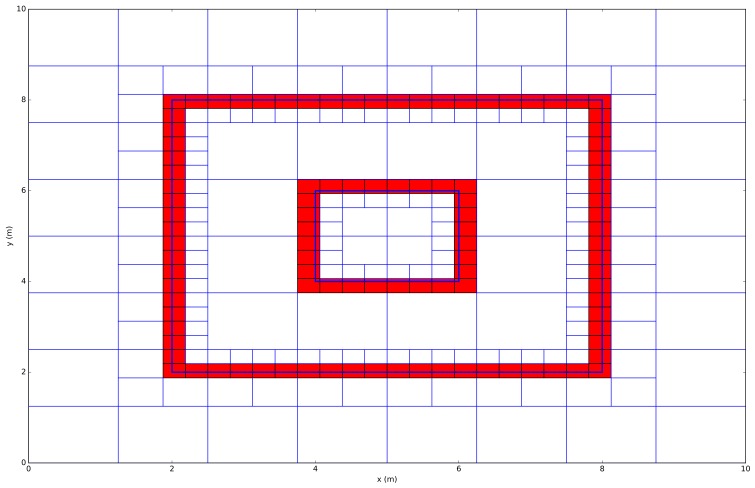
Example of a modeled environment in a Quadtree data structure. The red cell indicates the occupied cells.

Based on the type of the Quadtree, different nuances can be made in the implementation towards the minimum number of cells or maximum level of details. Therefore, different kinds of applications are possible. Firstly, a Quadtree that contains points is being applied in navigation applications in order, for example, search for shortest paths. Secondly, a region based Quadtree is used to improve the performance of either lossless and lossy compression techniques in image processing. Furthermore, in this paper, two Quadtree types are implemented. First, a Quadtree that contains points is used to construct line segments that are further inserted in the second Quadtree type that contains line segments. The main difference between both types is the insertion process of the object. For example, a Quadtree that contains points has to check whether a point is inside a square or not. Second, a Quadtree that contains line segments has to evaluate whether a line segment is inside or outside a square or if the line segment overlaps the square. Figure 3 visualizes an example of a line Quadtree where every red cell indicates the existence of a line.

## 4. Ray Launching Propagation Loss Model

**Figure 4:**
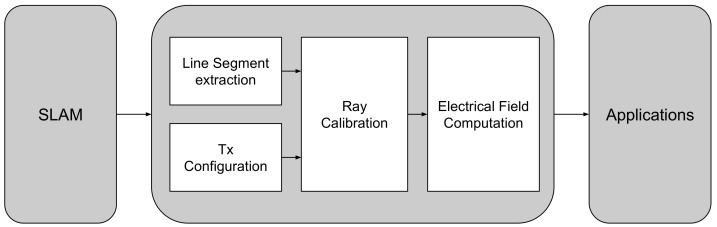
Overview of the ray launching propagation loss model.

The proposed ray launching propagation model is built upon four methods. Each method defines a specific task and requires the correct input in order to produce the appropriate output. An overview of the individual tasks is illustrated in Figure 4, which fits in the global perspective where the result of a SLAM algorithm is used by the propagation model. Subsequently, the result of this propagation model can be used for localization or coverage applications.

Since we are proposing a ray-launching propagation loss model that simulates the received signal strength and, more fundamentally, the electrical field based on an 2D map that was modeled with a SLAM algorithm, different assumptions need to be made. First, the environment model is 2D. This includes no reflections from ceiling and floors being included. Second, the thickness of the wall is not considered. Third, diffractions are not included and, finally, only vertical polarizations are assumed.

In this section, each method is described according to the mathematical background that is necessary for each method. Furthermore, the algorithmic implementation of each method is explained so that it is possible to indicate all contributions, which are proposed in the following overview of the different methods:

1. Line-segment extraction: This method, explained in Section 4.1, contributes to the extraction of line segments and the segmentation of walls and objects by applying a region growing connected component labeling algorithm on top of the Quadtree that contains points.
2. Device configuration: As described in Section 4.2, each device holds a simulation configuration that contains necessary parameters.
3. Ray Calibration: During this process, which is explained in Section 4.3, every ray will be launched according to a transmitter device configuration and will reflect and refract in the environment model that is defined by a Quadtree or an Octree. Because each environment model is modeled with a maximum resolution level, every ray will be launched according to that level. In order to increase the performance of this process, all rays are processed in a parallel fashion. The result of this ray calibration method is a list for every ray that exists of all visited cells. This list contains the total distance from transmitter to receiver together with the Fresnel reflection coefficients. Besides the fact that every ray is calibrated towards the maximum number of reflections and refractions, all binary addresses of the visited cells were stored in the object. This enables the usability to re-use this calibration data in order to simulate the signal strength for a different frequency or to apply a simulation where a subset of the rays is used, which makes it scalable and efficient to apply a benchmark.
4. *Electrical Field Computation*: Due to the previous calibration process, all parameters in order to compute the electrical field at every visited cell for each ray are known. Section 4.4 computes for each visited cell the electrical field related to these parameters. As a result, a coverage map can be created by computing the sum of all electrical fields at every cell that was visited.

Finally, this propagation model delivers a generic abstraction layer that makes it possible for applications to start, configure, and query the outcome of the simulation. Such a query can be the computation of the total electrical field, the received signal strength, the impulse response or the individual electrical field regarding each ray at a specific location in the environment model. For the sake of visualization purposes, a generic environment model is used in this section so that the different parts can be visualized in a clear way.

### 4.1. Line Segment Extraction

To extract line segments from individual points, a region growing connected component labeling algorithm is applied. Such an algorithm is able to cluster points based on local feature descriptors. In order to do this, a K-nearest neighbor (KNN) algorithm is required to find a nearest neighbor according to a specific location that is defined by a given seed location in an environment model *Q*.

To cluster the environment so that all occupied nodes that are part of the same wall can be distinguished, a classification is necessary. This includes the computation of a feature descriptor such as the principal components. Next, a KNN algorithm gets the nearest neighbor of the given seed location, which enables the comparison of the feature descriptors based on both Quadtree nodes.

This results in a method that is able to do the classification based on the eigenvectors that belong to the largest eigenvalues of the covariance matrix of both Quadtree nodes. Both eigenvectors are compared to each other by applying a threshold ϵ on the angle that is specified between the eigenvectors and the *x*-axis. Additionally, a region growing solution is implemented that will change the seed location according to the nearest neighbor. This process maintains a list of all visited nodes and stops when all occupied nodes are visited. Given the constraint of a regular office or room environment, the angle between walls is around 90${}^{\circ }$, which means that the eigenvectors of two walls are orthogonal to each other. Furthermore, because none of the corners are perfectly aligned in reality, two walls can be classified when both angles of one eigenvector related to the *x*-axis does not fit in the range of the angles of the other eigenvector that is computed with ϵ.

**Figure 5:**
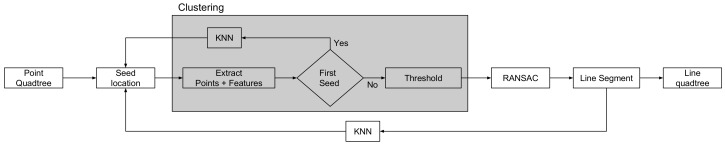
Region growing based line extraction.

The output of the algorithm is a Quadtree that contains lines and a list of clusters that indicates which line segments belong to each other and thus represents a wall or the same object. Figure 5 illustrates all different steps that are necessary to process the region growing connected component labeling algorithm, which is further elaborated in the following paragraph.

The initial Quadtree that exists of points is processed according to two main sub-algorithms: First, the region growing process maintains a list of location addresses that were visited and a list of the classified clusters. An initial seed location is manually defined. Next, the clustering process classifies the points of the closest neighbor in three steps. The implementation of this process follows the following steps:

1. The first step in the clustering process is the extraction of all environment points at location $P_{seed}$ by recursively visiting the environment model. Furthermore, the principal components will be computed for these points. In the case of the first seed location, the address of the nearest neighbor node will be added to the list of seed locations by applying the KNN algorithm.
2. The clustering process defines if both datasets are part of the same cluster or not. This process uses a predefined threshold ϵ. In the case of the simplified example, this threshold was configured to $90^{\circ }$.
3. The region growing process selects the next nearest neighbor node in order to cluster the new node. This process keeps searching for nodes that contain environmental points until all nodes are visited. Finally, line segments have to be created based on the clusters that were found. To do this, two approaches are possible:
  (a) a RANdom SAmple Consensus (RANSAC) algorithm, which automatically excludes outliers, so that the optimal line segment can be found regarding the points of each cluster. One of the advantages of this approach is that an optimal line segment will be found according to the given points. When the number of points is low, it is hard to get an optimal line segment since RANSAC needs to sample random samples of different point pairs.
  (b) connecting the centroids of the set of points of each cluster. The main disadvantage of this approach is that the number of clusters has to be large so that it performs an accurate segmentation.

**Figure 6:**
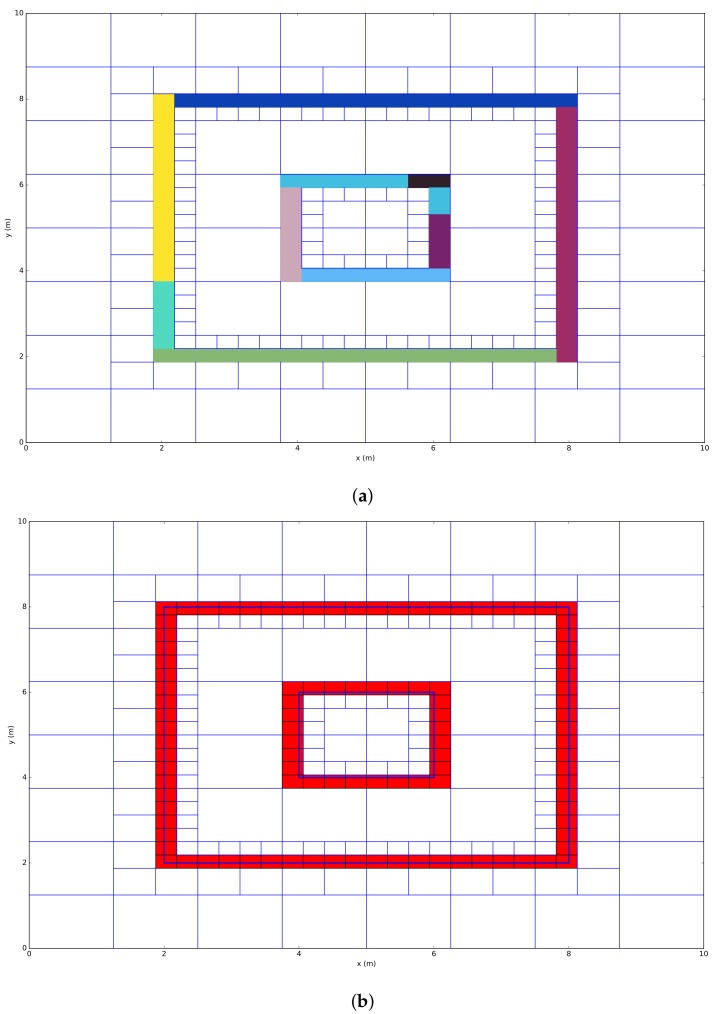
Clustering + line segment extraction. (**a**) overview of region growing based line extraction; (**b**) line extraction.

As a conclusion of the line segment extraction algorithm, an example is visualized in Figure 5, which indicates the classified clusters in Figure 6a and the line-segments in Figure 6b. In this example, the first seed location was configured at location $\left\{2,3\right\}$. In order to use this model to calibrate each ray, each cluster needs to have permittivity. This value is assigned in a supervised fashion through iteration.

### 4.2. Device Configuration

Because every simulation starts with a specific purpose and a certain expectation of what the result should be, a good configuration is necessary. Such a configuration contains different parameters about the environment model, transmitter devices, receiver devices, and antennas. This means that a priori knowledge about the propagation model is required in order to get a correct result given the input data. Furthermore, as a result of the application that is specified, the conclusions will be different. For example, localization applications that use the Angle of Arrival (AoA) requires the electrical field to be a complex number since it includes phase information, whereas traditional RSS localization uses the Received Signal Strength, which is expressed in dBm. A general configuration contains the following parameters:

- Transmitter location $\left\{x,y,z\right\}$,
- Transmitter power (dBm),
- Transmitter antenna gain (dB),
- Radiation pattern (monopole, dipole),
- Number of rays,
- Maximum level of the reflection tree,
- Receiver location $\left\{x,y\right\}$,
- Receiver antenna gain (dB),
- Maximum level of the environment model,
- Environment model.

### 4.3. Ray Calibration

The core of this propagation model is based on the principle of ray launching. These rays are launched in different directions specified by an antenna radiation pattern. After the rays are generated, each ray will be inserted in the environment model at the lowest level of the environment model so that the space between the transmitter and the ray endpoint can be addressed and traced. Because of the fact that each ray is able to reflect and to refract in the environment when it intersects with an environment segment, each ray needs to be traced using a depth-first traversal algorithm. This traversal algorithm facilitates the process of maintaining a list that holds the total distance between the visited cell and the transmitter location, the Fresnel reflection coefficients, and the binary address of each visited cell. Thus, each time that a ray intersects with a line segment of the environment, the reflection and refraction parameters are computed. When $P_{reflection}$ is defined, a reflection is computed and a new ray is traced until $depth_{current}$ is equal to the maximum reflection value, which is given by the configuration. Next, a refraction ray is computed and traced.

**Figure 7:**
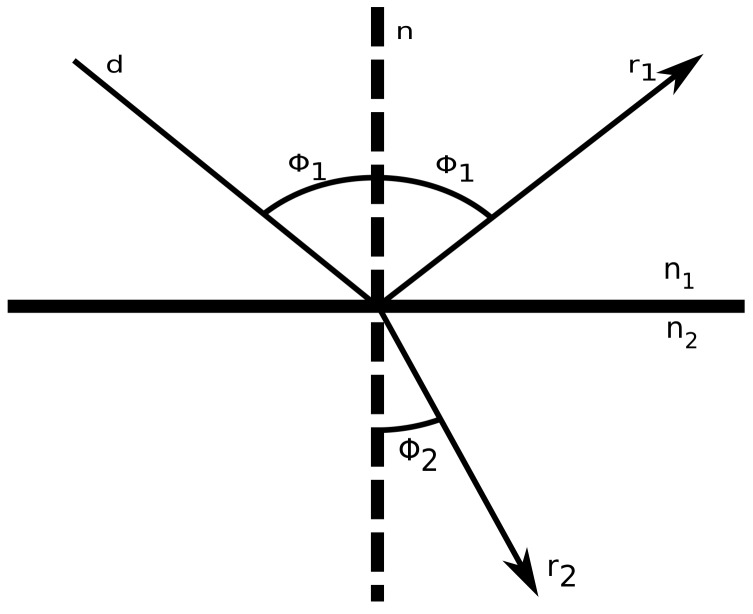
Reflection.

Moreover, the estimation of a reflection and a refraction is divided into two steps: first, the computation of the reflection angle is computed according to Fermat’s principal, which states that the angle of reflection is equal to the angle of incident $\theta_{1}$, whereas the reflection vector **r** can be computed with the following Equation (1):

(1) r=d-2(d·n)n,

where d·n is the dot product of the normalized incident vector *d* and the normalized normal vector n of the environment line segment. The normal vector is computed according to the following $(-\left(y_{1}-y_{2}\right),$$\left(x_{1}-x_{2})\right)$ formula. The second step assumes Snell’s law to compute the transmission angle that appears when a ray refracts. This law defines the ratio between the incident angle and the transmission angle according to the refractive index *n* of the material it is penetrating. This correlation is defined with the following formula:

(2) $$\frac{\sin(\theta_{1})}{\sin(\theta_{2})}=\frac{\eta_{2}}{\eta_{1}},$$

where $\theta_{1}$ and $\theta_{2}$ are the incident and transmission angles, respectively. Furthermore, $\eta_{1}$ and $\eta_{2}$ are the refractive indexes of the specific material as illustrated in Figure 7. In most common applications, $\eta_{1}$ is the refractive index of air, while $\eta_{2}$ can be any material such as wood, concrete, plasterboard, metal, stone, etc. This refractive index can be further explained according to the following equation:

(3) $$\eta =\sqrt{\epsilon \mu },$$

where ϵ denotes the permittivity of the material, which is described as $\epsilon =\epsilon_{r}\epsilon_{0}$, where $\epsilon_{r}$ is the relative perimittivity and $\epsilon_{0}$ the absolute permittivity. This absolute permittivity or the permittivity of free space is measured in vacuum and is approximately 8.854×$10^{-12}$ F/m. On the other hand, the permeability μ can be described as $\mu =\mu_{r}\mu$${}_{0}$, where $\mu_{r}$ is the relative permeability of a material and expresses the influence of the material on the wave resulting from its magnetic properties. Because most of the materials become non-magnetic when the frequency is higher then 1 MHz, the relative permeability is 1 and thus Equation (3) becomes $\eta =\sqrt{\epsilon }$ [28]. For this reason, the material parameter of each material in the environment model includes only the permittivity since the propagation model is simulating the electrical fields at frequencies higher then 1 MHz.

Furthermore, to describe the amount of power that will be lost due to reflection or transmission, both incident and transmission angles are used to compute the Fresnel coefficients. These Fresnel coefficients describe the correlation between the reflectivity, the angle of incident and the polarization, and the computation of reflection (Γ) and transmission (*T*) related to a vertical polarization. These coefficients are computed by the following Fresnel equations:

(4) $$\begin{array}{c} \Gamma_{⊥}=\frac{\eta_{1}\cos\left(\theta_{1}\right)-\eta_{2}\cos\left(\theta_{2}\right)}{\eta_{1}\cos\left(\theta_{1}\right)+\eta_{2}\cos\left(\theta_{2}\right)}, \end{array}$$

(5) $$\begin{array}{c} T_{⊥}=\frac{2\eta_{1}\cos\left(\theta_{1}\right)}{\eta_{1}\cos\left(\theta_{1}\right)+\eta_{2}\cos\left(\theta_{2}\right)}, \end{array}$$

where $\left\{\theta_{1},\theta_{2}\right\}$ corresponds to the incident and transmission angles.

**Figure 8:**
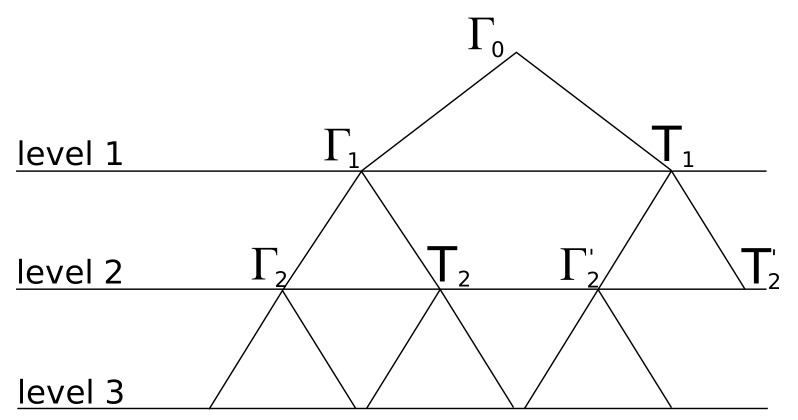
Reflection binary tree data structure + ray order illustration.

**Figure 9:**
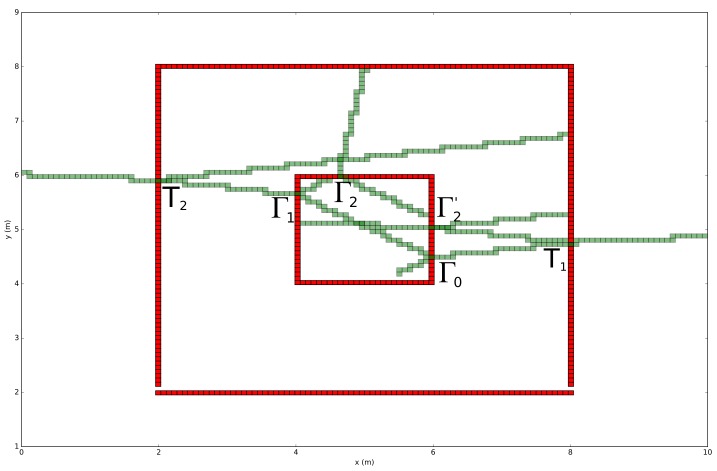
Example of a ray calibration.

As previously stated, a depth-first traversal algorithm is implemented to trace each ray in the environment until a pre-configured level of depth is reached. The meaning of this pre-configured level applies to the number of recursions that each ray can take. To illustrate this, Figure 8 shows a binary tree data structure that indicates all reflection and refraction interactions of the ray that is visualized in Figure 9 starting from location $\left\{5.5,4.2\right\}$. This traversal algorithm will process the illustrated ray, which is defined by two points $P_{1},P_{2}$, in environment model *Q* as follows:

1. A depth-first search algorithm that recursively traces the Quadtree data structure until location $P_{1}$ fits within the range of the node.
2. An algorithm that keeps track of the visited cell list and ensures that the recursion level of the reflection is smaller then the pre-configured level. Because of the fact that each ray is inserted into the environment model by splitting each ray in small line segments with a permittivity of 1, an intersection point between an environment line segment and a ray segment can be computed with traditional mathematics. This intersection only occurs when the Quadtree node contains an environment object. Since each reflection and transmission is traced in a recursive fashion, the following Figure 8 illustrates the recursion order that each ray undergoes. At the first intersection $\Gamma_{0}$, a reflection $\Gamma_{1}$ and transmission $T_{1}$ occurs. Second, because of the depth first implementation, $\Gamma_{2}$ is the next reflection intersection. Since $\Gamma_{2}$ appears at level 2 and the pre-configured level is equal to 3 this ray will be traced in the direction of the reflection angle and will stop at the following intersection. Subsequently, the returning recursion will trace $\Gamma_{2}$ in the direction of the refraction angle. Third, as a result of the recursive implementation, the intersection at refraction $T_{2}$ is found and traced. Finally, the other reflections and refractions are found and traced in a similar approach.
3. Detecting if the ray is part of the current Quadtree node. This process includes the estimation where the ray segment denoted by $P_{1}$ and $P_{2}$ is crossing the axis- aligned bounding box defined by $P_{min}$ and $P_{max}$. Since each ray segment can be expressed as y=mx+b, where *m* denotes the slope and *b* the location where the segment crosses the *y*-axis. On the other hand, an edge of an axis-aligned bounding is always parallel with one of the coordinate axis. This means that y=mx expresses a line that is parallel with the *x*-axis. In order to check if the ray crosses this Quadtree node, $P_{min}$ and $P_{max}$ needs to be incorporated so that all line equation that refers to the edges of the bounding box are crossing with the ray segment or not. In case when a ray is crossing any ray segments, the algorithm increase the ray segment so that $P_{1}$ is located in the next Quadtree cell. This makes it possible for the next recursion to locate the next Quadtree node that is part of the ray.
4. Since the traversal algorithm traverses a ray in a Quadtree data structure according to a depth first search algorithm to localize each node, a tail recursion is applied that keeps the ray traversal algorithm running until the end of the ray is reached or the outer boundaries of the environment model are reached.

Because of the independence of each ray, this process can be executed in parallel, which will increase the performance of a single simulation as can be seen in Section 6. The implementation of this solution is based on the generation of a single environment model that is used to calibrate each ray. Subsequently, a temporary copy of this environment model is used to trace every ray.

### 4.4. Electrical Field Computation

The objective of calibrating the rays in the environment model delivers the possibility of computing the electrical field according to a specific simulation configuration. In addition to Section 4.2, a configuration can be extended so that it can contain a range of frequencies or a different number of maximum reflections that a ray can reflect and refract. As a result of the ray calibration process, a list for each ray is created that contains all the addresses of the visited environment cells together with the computed reflection coefficients. Furthermore, this list is used to compute the electrical field at each visited location according to the following Equation (6):

(6) $$E_{x,y}=E_{0}\frac{e^{-jkd_{x,y}}}{d_{x,y}}\prod_{g=1}^{n}\Gamma_{g}\prod_{h=1}^{m}T_{h},$$

where $d_{x,y}$ defines the total distance between location x,y and the transmitter location. When one or more reflections occur, this total distance is computed as the sum of the distance between location x,y and the last reflection and the distances between all reflections and the transmitter location. Subsequently, this distance represents the total length of the ray. Furthermore, *n* and *m* defines the number of reflections Γ and refractions *T*, respectively. In accordance with Equation (6), a multiplication of the calibrated reflections and refractions is required in order to compute the electrical field at location x,y relative to the transmitter location. Next, $E_{0}e^{-jkd_{x,y}}$ specifies the complex electrical field related to the transmitted power, the total distance and the frequency. The parameter *k* in this formula defines the wave constant $\frac{2\pi }{\lambda }$ and $E_{0}$ is the reference electrical field computed according to the following Equation (7):

(7) $$E_{0}=\sqrt{\frac{\eta_{0}}{4\pi }\cdot P_{t}G_{t}},$$

where $\eta_{0}$ is the intrinsic impedance of free space or 120π. $P_{t}$ is the transmitted power and $G_{t}$ is the gain of the used transmitted antenna.

### 4.5. Applications

In order to use this propagation loss model in applications such as wireless coverage optimization or wireless localization, a total electrical field needs to be computed according to Equation (8):

(8) $$E_{tot}=\sum_{p=1}^{q}E_{p}.$$

In this formula, *q* is the number of the individual electrical fields $E_{p}$ at location x,y. Due to the computation of the electrical field at location x,y for every ray, all electrical fields with the same reflection location can be neglected and need to be removed from the list. This list includes all the electrical field of every ray that has traversed the individual node during the calibration process, which can be summed to obtain the total electrical field. As a result of applying the sum, constructive and destructive waves behaviors are included because of the presence of the phases. Moreover, when a received signal strength (RSS) is necessary, the total electrical field has to be converted into a power density that is relative to the intrinsic impedance of air. This conversion can be computed with the following Equation (9):

(9) $$P_{r}=\frac{E_{tot}^{2}\lambda^{2}G_{t}G_{r}}{\eta_{0}4\pi }.$$

**Figure 10:**
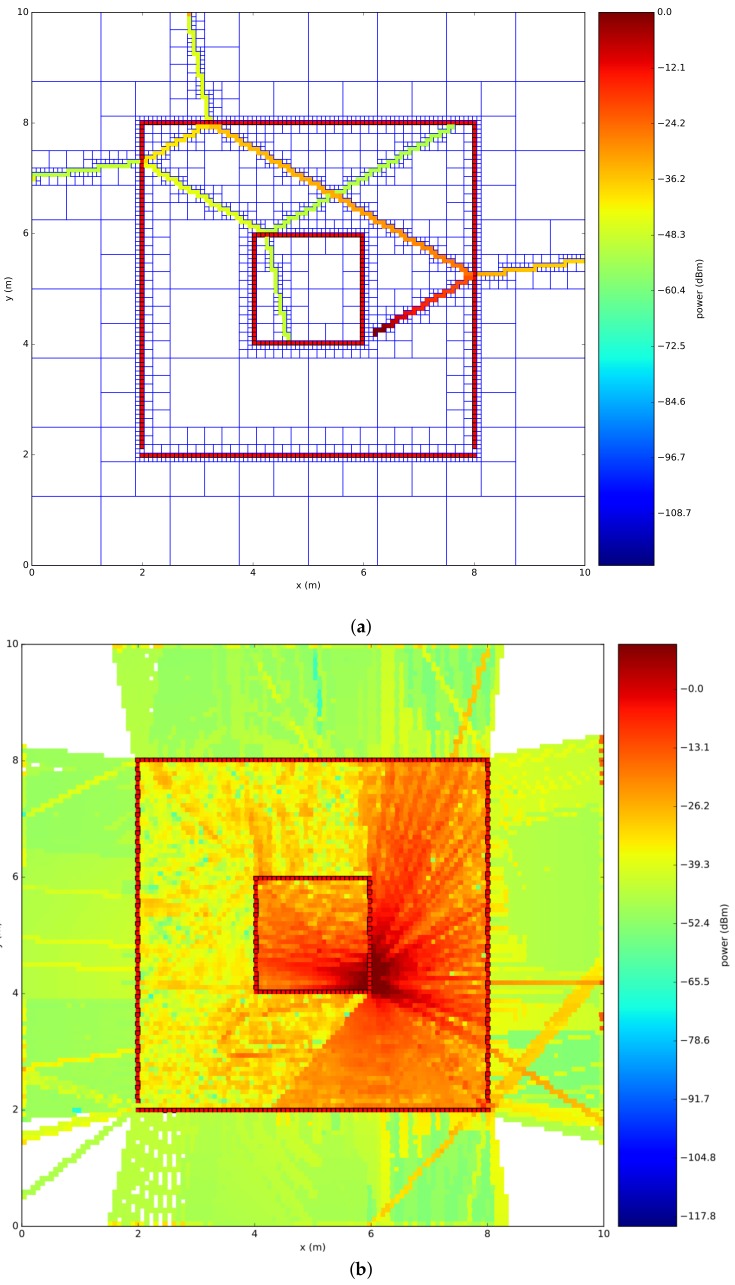
Example single ray propagation. (**a**) one ray; (**b**)example of 1000 rays.

Figure 10a shows the results of the computation of the total electrical field for one ray at every location that was calibrated for one ray, while, on the other side, Figure 10b shows the results of the total electrical field converted into a power density, when 1000 rays are launched in the environment model.

In addition to applications that are applied in indoor environments, the term multipath is often used to indicate the influence of different propagations phenomena that a ray undergoes. This can be described more in detail as the influence due to reflections, refractions, and diffractions. Since each location contains a list of all electrical fields of each ray that traversed the specific node, every distance can be converted in a time *t*, which indicates the duration of each ray to travel from transmitter to location x,y. This conversion is computed by dividing the distance with the speed of light as follows:

(10) $$t=\frac{d}{c},$$

where *d* is the distance that was used to compute the electrical field and *c* is the speed of light. As a result of this approach, an approximation of the impulse response at location x,y is possible. Because of the deterministic flavor of the ray-launching propagation model, the approximation can be reached when the number of rays is very high.

## 5. Materials

In order to prove the objective of simulating the signal strength in a realistic way, a validation approach is necessary. To validate our propagation loss model, different aspects are analyzed and evaluated regarding real measurements that were taken in two different indoor office environments. This section first explains the general validation model in order to validate each simulation so that the different results can be analyzed and evaluated in the same way. Second, two test environments and the respective hardware is explained that was used to take all the measurements.

### 5.1. Validation Model

**Figure 11:**
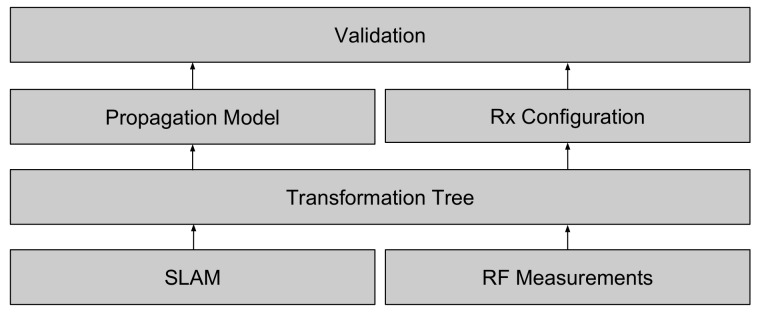
Overview of the validation approach.

This automated validation model is based on four layers as can be seen in Figure 11.

The first layer specifies on one side the SLAM algorithm that enables it to localize a robot relative to a map that is created by sensing the environment with a laser that was mounted on the robot. On the other side, radio frequency measurements are taken at different locations with a DASH7-receiver that was mounted on the robot. As a result of different losses due to passive components, a calibration measurement that is measured in the antenna’s far field is necessary. To incorporate this calibration measurement, the transmitted power is being calculated according to the *Friis Transmission equation*, which is then used in Equation (7). Second, a transformation tree is maintained during the measurement process. This transformation tree includes all geometric transformations between all transmitters that were placed in the environment and the first robot location. Next, as a result of this tree, the propagation model is able to simulate the total electrical field relative to each transmitter configuration and regarding the environment created by the SLAM algorithm. Such a transmitter configuration exists of the transmitted power, antenna gain, the antenna radiation pattern, the number of rays, and the frequency that was used. As a second result of the transformation tree, each receiver location can be extracted because, at every receiver location, a specific time delay of was applied. Lastly, a validation towards a specific transmitter configuration can be made by computing the accuracy in terms of the Root Mean Square Error, the Mean Absolute Error and the Mean Error between the measured signal strength and the predicted signal strength computed at the same location. To analyze both the correlation between the number of rays versus the environment resolution and the level of the reflection tree, different transmitter configurations have to be validated. This makes it possible to evaluate the result. Because of the generic implementation, the list of the rays that includes all the results can be used to validate a different simulation. For example, a simulation that holds the result of 6400 rays makes it possible to analyze the result of a simulation where 3200, 1600, 800, 400, 200, and 100 rays are launched. This will decrease the computation time that is necessary to evaluate the individual validations.

### 5.2. Test Environments

**Figure 12:**
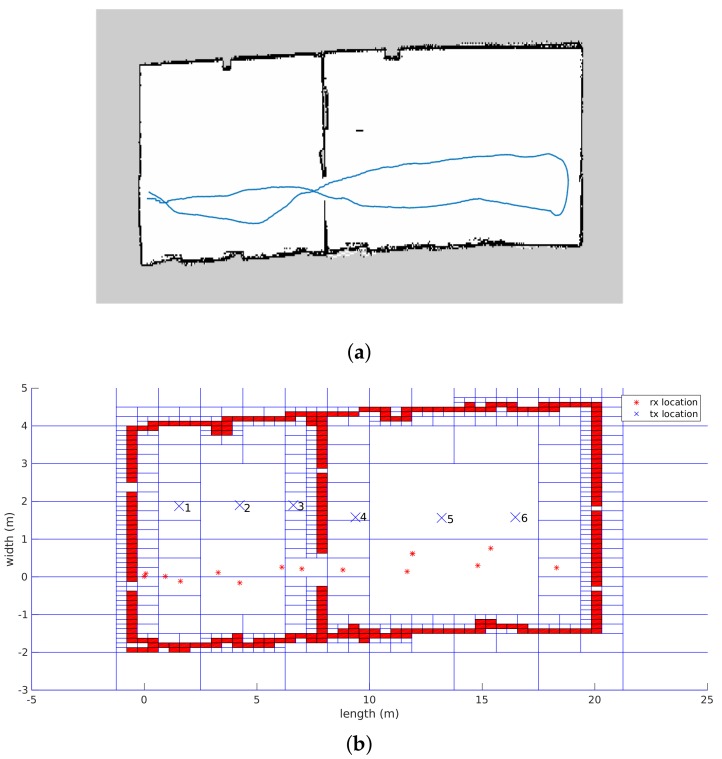
Office environment that was used to validate the propagation loss model. (**a**) SLAM results; (**b**) environment model with transmitter and receiver locations.

As stated before, the propagation loss model is validated according to two indoor office environments. The first environment is situated in the center of Antwerp. The environment consists of two adjacent rooms that are divided by a plasterboard wall that has a door. Both rooms were empty during the measurements where six transmitters were placed in both rooms at fixed locations, which are indicated in Figure 12b as blue crosses. The dimensions of the first room are 8.6 m × 6 m, whereas the dimensions of the second room are 12 m × 6 m. Next, the robot performed the measurements on 16 different locations that are illustrated in Figure 12b with red stars. Subsequently, 96 individual links can be validated. On the other hand, simultaneously with the radio measurements, the robot processes the measurements according to a SLAM algorithm so that an environment map is created. The result of this map, modeled as a Quadtree with a resolution up to level 8 (0.234 m × 0.078 m) and the trajectory of the robot, can also be seen in Figure 12.

**Figure 13:**
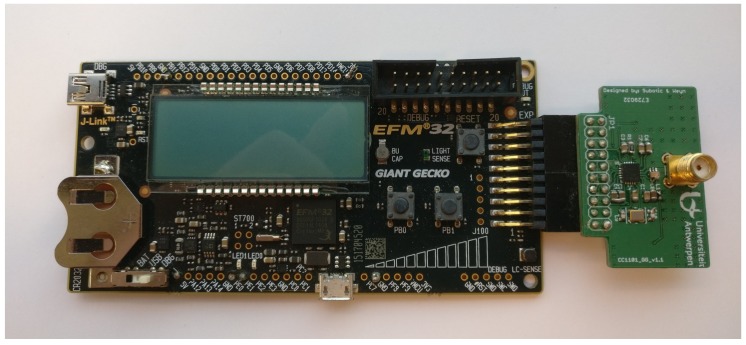
Hardware that is used in the Campus Paardenmarkt (CPM) environment for transmitter and receiver.

To validate the propagation model, radio measurements at different locations during one minute on one sub-1GHz frequency, 434.56 MHz were made. During this period, all the received signal strength values that were sent every second from our sub-1 GHz low power transmitter nodes were captured together with the laser and wheel odometry data. On the one hand, the embedded hardware that we used for this environment was the CC1101 radio chip of Texas Instruments in combination with the Giant Gecko development kit of SiLabs as can be seen in Figure 13. In addition to the micro-controller and the radio chip, a monopole antenna is used in order to emit the signals on the medium. This monopole antenna has a peak gain of +3 dBi and is matched for 433 MHz. On the other hand, the low power mid-range DASH7 open source stack was used to program the embedded hardware [15].

**Figure 14:**
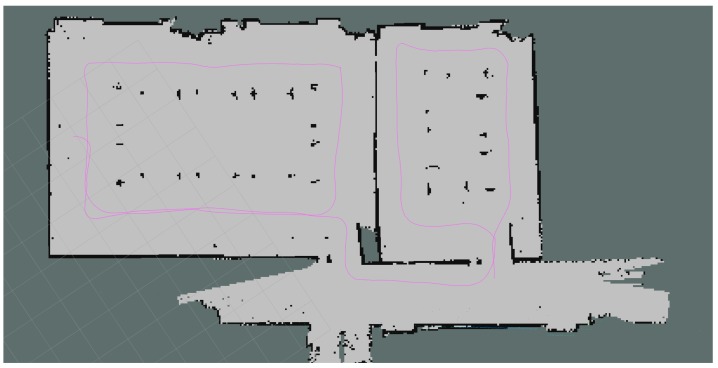

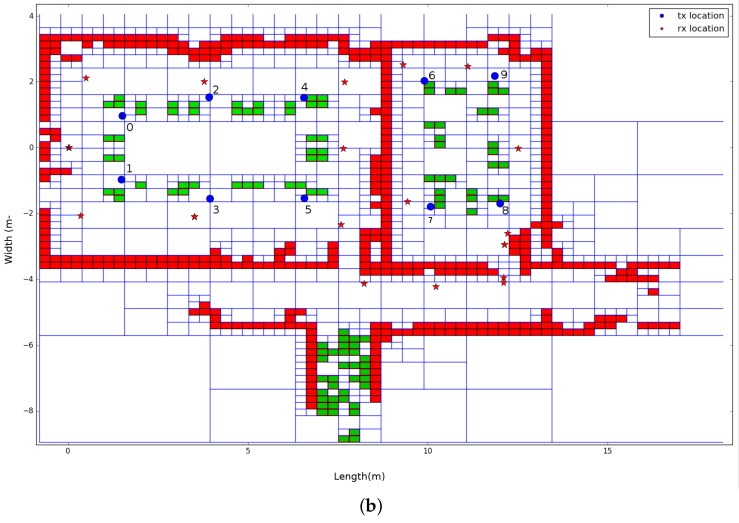
iGent Tower. (**a**) SLAM results; (**b**) environment model with transmitter and receiver locations.

The second environment that was used to validate the propagation loss model is located in the iTower in the city of Gent. This environment is also divided by two rooms, one large conference room and another smaller meeting room. Both rooms were equipped with the regular conference and meeting room equipment such as tables, chairs, and projector. Subsequently, the dimensions of the large conference room is 9.4 m × 6.8 m and the small meeting room is 4.6 m × 6.8 m. As a result of the realistic modeling solution, the Quadtree that is modeled on resolution 8, which results in a minimum cell size of 3.6 cm × 2.6 cm, can be seen in Figure 14b. Together with the environment model, the trajectory, the location of 10 transmitters and 20 receivers are illustrated, which result in 200 individual links to be validated. In addition to this, the robot was equipped with four receivers, so these 200 individual links can be multiplied by 4 so that the total number of links that can be used to validate the propagation model is 800 links.

**Figure 15:**
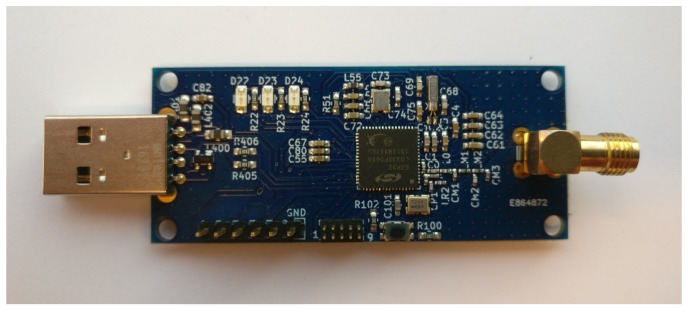
Hardware that is used in the iTower Gent environment for transmitter and receiver.

The hardware that was used in this environment that makes a validation of the propagation model possible is based on a System-on-the-chip (SoC) design of SiLabs that integrates the Si4460 radio chip with a Leopard Gecko Micro-controller, which is based on the ARM Cortex-M3 architecture. This SoC is the core component of the DASH7-USB design that can be seen in Figure 15. Furthermore, every link that will be evaluated consists of measurements that were captured during one minute at a receiver location. Because of the wireless aspect, a monopole antenna that is matched for 433 MHz is used. This antenna has a maximum peak gain of +3 dBi.

## 6. Results and Discussion

This section presents the individual results of both office environments and the discussion of these results. The two environments are evaluated in the same way according to the general validation model, which is explained and discussed in Section 5. In order evaluate this propagation loss model, four statistical estimators are used to quantify the performance of the propagation loss model. First, the mean error is used to quantify if the propagation model is performing better or worse than what was measured. The Mean Error $E_{ME}$ is computed according to the following equation:

(11) $$E_{ME}=\frac{1}{n}\sum_{i=1}^{n}P_{measured}-P_{simulated},$$

where $P_{measured}$ is the average signal strength that was measured at a receiver location *n* during a period of one minute and $P_{simulated}$ is the signal strength that was simulated at the same receiver location *n*. The second estimator that is used to express the performance of the propagation loss model is the Mean Absolute Error $E_{MAE}$. This estimator is mainly used to find the average error and is calculated with the following equation:

(12) $$E_{MAE}=\frac{1}{n}\sum_{i=1}^{n}\left|P_{measured}-P_{simulated}\right|.$$

The third estimator that is applied to illustrate the performance of the propagation loss model is the Root Mean Square Error $E_{RMSE}$. This estimator is widely used to indicate the performance of system and is computed in Equation (13). Since it is sensitive to outliers, this value is not always the best option:

(13) $$E_{RMSE}=\sqrt{\frac{\sum_{i=1}^{n}{\left(P_{measured}-P_{simulated}\right)}^{2}}{n}}.$$

Finally, precision is used to quantify the standard deviation of the errors. These estimators will be used to evaluate each environment in four different perspectives:

1. By analyzing the correlation between the number of rays, the resolution of the environment, and a specific level of the reflection tree, the best global accuracy in terms of RMSE of all individual links is evaluated.
2. The influence of the reflection tree level or changes in small scale fading is analyzed by simulating a different set of rays related to the RMSE according to different resolution levels of the environment.
3. The accuracy of all transmitters is evaluated by analyzing the differences between the simulated signal strength and the measured signal strength that was measured during one minute at the different receiver locations in terms of the ME, the MAE, the RMSE, and the precision.
4. The performance of the propagation loss model is analyzed related to the number processing units and the resolution of the environment. Furthermore, this analysis takes the optimal level of the reflection tree and the optimal number of rays as input.

### 6.1. Office Environment 1

The first office environment exists of 221 line segments and contains six transmitters that operate at 433 MHz. Furthermore, 16 different locations were used to receive DASH7 messages that were sent periodically. This results in 96 links that are used to evaluate the ray launching propagation loss model. In the following sections, the results of the four perspectives in order to evaluate the propagation models are shown.

#### 6.1.1. Resolution

**Figure 16:**
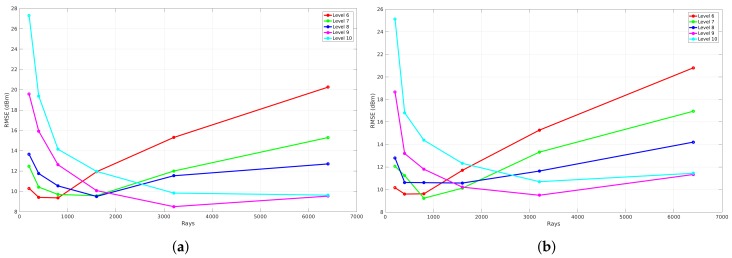
RMSE validation with phases shifts included of office Environment 1. (**a**) zero neighbors are included; (**b**) eight neighbors are included.

In order to analyze the correlation between the amount of rays that are launched and the global accuracy in terms of RMSE, the resolution of the environment model is changed. Because of the spatial data structure modeling technique, an environment can be described according to a specific cell size. This results in the possibility to represent an environment with a lot of details or without any details, which influences the global accuracy when validating the propagation loss model. Figure 16a visualizes the global accuracy on the *y*-axis and the number of rays on the *x*-axis. This figure shows the results of a set of simulations that were computed according to a set of resolution levels ranging from level 6 to 10 and a set of rays (200, 400, 800, 1600, 3200, 6400). As can be seen in the figure, the global accuracy of a simulation at resolution level 6, represented by the red line, first decreases to a minimum when the number of rays increases. Furthermore, when the number of rays further increases, the global accuracy gets worse. In contrast to this observation, the global accuracy of a simulation at resolution level 10, represented by the cyan line, converges to a minimum when the number of rays increases. In addition to this trend, each resolution level has a different minimum.

**Figure 17:**
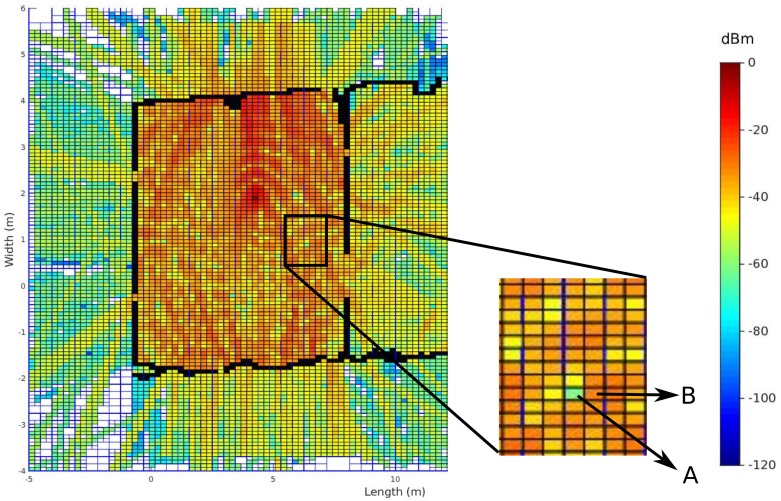
Local constructive and destructive phenomena.

**Figure 18:**
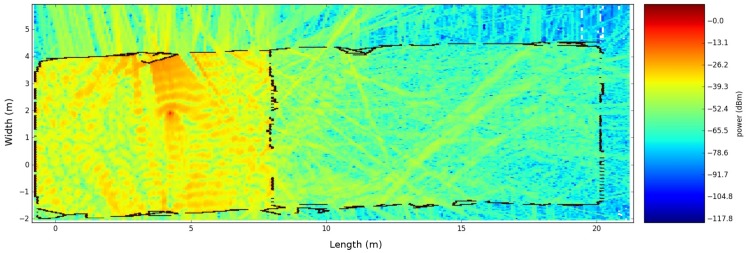
Received Signal Strength (RSS) heatmap of the transmitter that is located at position 2.

As a result of these observations, two principles are further analyzed in order to get a more fundamental understanding of these results. The first principle covers the inclusion of the neighborhood of the cell that is used for retrieving the simulated signal strength. In order to analyze this principle, the RMSE is calculated based on the difference between the measured signal strength and the simulated signal strength at the specific cell. In case the neighborhood is included, the average signal strength of the specific cell and the eight surrounding cells are used to compute the RMSE. Moreover, as a result of reflections, the phase of the electrical field shifts so that constructive and destructive phenomena occur. This can lead to local changes when a fine resolution is applied. An example of such local changes can be seen in Figure 17, where the highlighted surface that is part of the heatmap is visualized by Figure 18, which has different cells that differ a lot in signal strength compared to others. Thus, the signal strength of the cell that is indicated by A is much less than the neighboring cell that is indicated by B.

The results of this evaluation for the first office environment can be seen in Figure 16, where Figure 16a illustrates the validation where no neighbors are included and Figure 16b illustrates the validation when the eight surrounding neighbors are included. The main difference between both figures is the fact that the RMSE when zero neighbors are included is only 3 to 5 dB lower than the results where eight neighbors are included.

**Figure 19:**
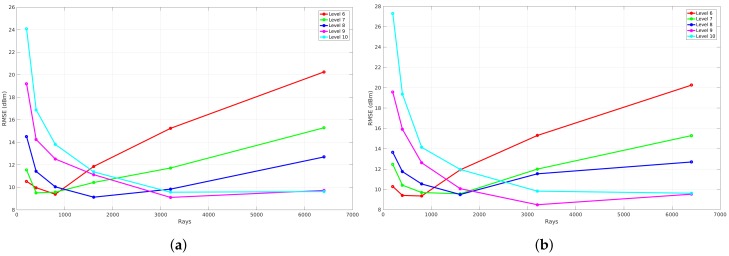
RMSE validation of the first office environment where eight neighbors are included. (**a**) no phase shifts are included; (**b**) phase shifts are included.

The second principle that is analyzed covers the idea of removing the phase shifts that are introduced by any reflection. This means that only the constructive behaviour is applied. Thus, the total electrical field, which is used in Equation (9), is described as the sum of the individual electrical fields that were computed in a cell by each ray that traversed that specific cell. When the absolute value of each individual electrical field is used to compute the total electrical field, no phase shifts are included. The same observation can be made as previously. Figure 19a where phase shifts are included gives a result where the RMSE is 3 to 5 dB worse than Figure 19b, where no phase shifts are included when the number of rays are limited to 1600.

#### 6.1.2. Reflections

**Figure 20:**
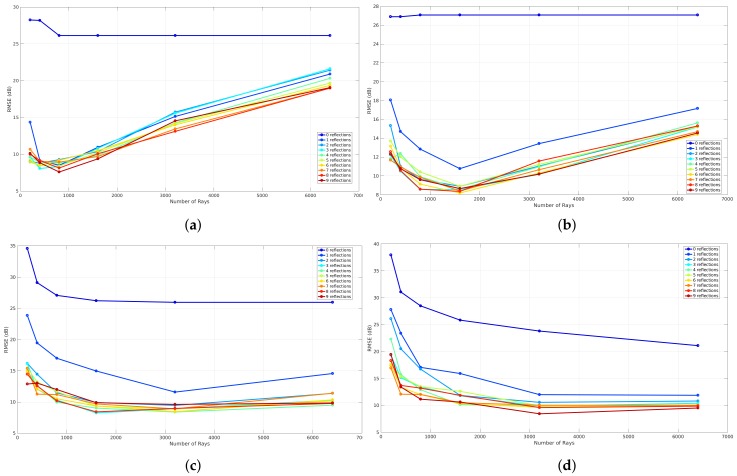
Reflection benchmark for office environment 1. (**a**) reflection benchmark at level 6; (**b**) reflection benchmark at level 7; (**c**) reflection benchmark at level 8; (**d**) reflection benchmark at level 9.

To evaluate the influence of reflections, a benchmark is applied that analyzes the global accuracy of a validation that is simulated with a specific resolution level. This results in four graphs that can be seen in Figure 20. Each graph represents the benchmark of a validation where different reflection recursion levels are applied at a specific resolution level. These graphs illustrate the number of rays that were used to validate each reflection recursion level on the *x*-axis and the RMSE expressed in dB on the *y*-axis. The meaning of the reflection recursion level is previously explained in Section 4.3 and is visualized in Figure 8. Thus, each reflection recursion level represents the lowest level of such a reflection tree. As a result of the correlation between the number of rays and the RMSE when different resolution levels are applied, the validation in order to evaluate the influence of the reflections is based on the result where zero neighbors are included and the phase shifts are allowed.

Figure 20 shows the results of such a reflection benchmark of this environment that is modeled at resolution level 6 (34 cm ×12.5 cm), 7 (17 cm ×6.25 cm), 8 (8.6 cm ×3.12 cm), and 9 (4.3 cm ×1.56 cm). Since this benchmark validates every simulation according to the environment model, a material parameter needs to be assigned in order to evaluate the influence of the reflections recursion level and the number of rays. Due to the fact that all the walls were the same material in this environment, one material parameter ϵ is assigned for all components, which was set to 4 since it was dry brick walls. Furthermore, nine different reflection recursion levels are evaluated. From these graphs, it can be seen that, when no reflections are configured, the RMSE is large and not converging when the resolution level is 6, 7. In the case when a reflection recursion level of 1 to 9 is configured, the RMSE is converging where the resolution levels are equal to 8 and 9. Moreover, when the resolution levels are equal to 6 and 7, the result of the RMSE first decreases towards an optimal number of rays. Second, when the number of rays further increases, the RMSE gets worse. This behavior shows that the cell size at resolution levels 6 and 7 is too large when the number of ray is larger than 800 for resolution level 6 and 1600 for resolution level 7. As the evaluation shows, a good trade-off can be found at a reflection recursion level of 5 since the RMSE is not improving when the reflection recursion level is larger.

#### 6.1.3. Validation

**Figure 21:**
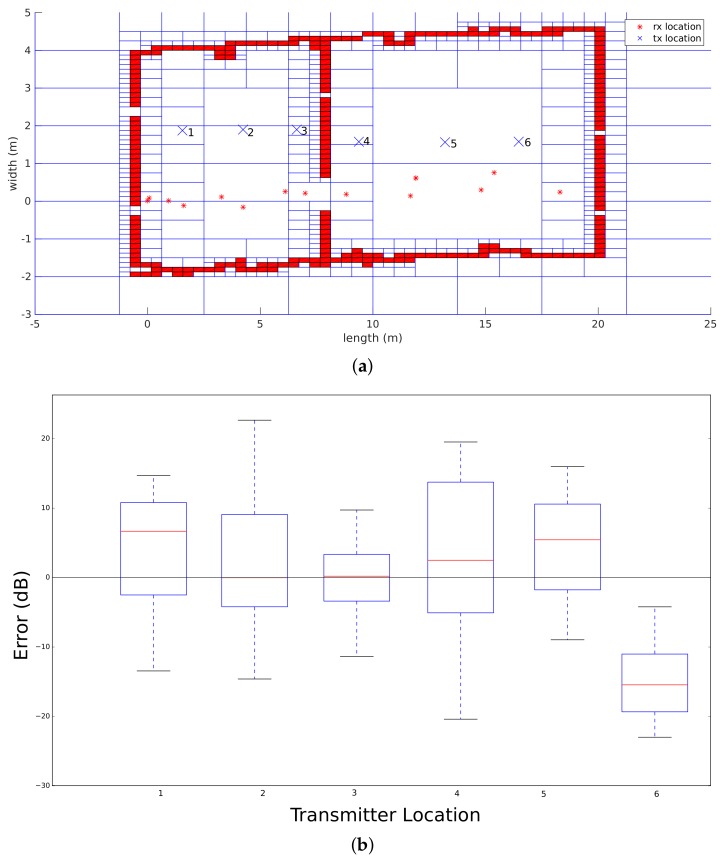
Office environment model 1. (**a**) environment model with transmitter and receiver locations; (**b**) validation of the different transmitters.

As shown in Figure 12, the result of the SLAM approach can be used to extract the line segments in order to assign a material parameter to each line segment. As a result of the line segment extraction process, all line segments are inserted into a Quadtree data structure as can be seen in the following Figure 21a, where the transmitters are indicated by the blue crosses and the receiver locations by red stars. Subsequently, this Quadtree is modeled with a resolution level of 8. This means that each cell has a width of 8.6 cm and a height of 3.1 cm.

Next, to evaluate the propagation loss model, the correlation between the different parameters needs to be analyzed. As a result of this analysis, best results for this environment are retrieved by applying a simulation on resolution level 9 and 3200 rays. Furthermore, the best recursion level of the reflection tree was found at level 5. Next, the transmitted power $P_{tx}$ was configured by computing the transmit power according to the Friis Transmission equation and a calibration measurement that was taken in the far-field. The gain $G_{tx}$ and $G_{rx}$ was configured to −5.6 dBi. This value was measured in an anechoic chamber, where we could measure the real output power without interference of reflections. Furthermore, with a receiver, we were able to receive a signal from the transmitter, which made it possible to calculate both antenna gains and extra losses due to passive components according to the Friis Transmission equation. This simulation results in the following figure, where all differences between the average received signal strength that was measured and simulated are illustrated regarding each transmitter.

This result is evaluated by computing the global accuracy and precision of all transmitters. As stated before, the global accuracy is described by four parameters that use the error between the average signal strength that was measured and simulated. The accuracy of each simulation that was performed for every transmitter can be seen in Table 1.

Figure 21b described the results of all receiver locations related to each transmitter. Other results of the propagation loss model can be seen in Figure 18, which visualizes the signal strengths in the environment of the transmitter that is located at position 5. The global accuracy of this office environment can be found in Table 2.

#### 6.1.4. Performance

**Figure 22:**
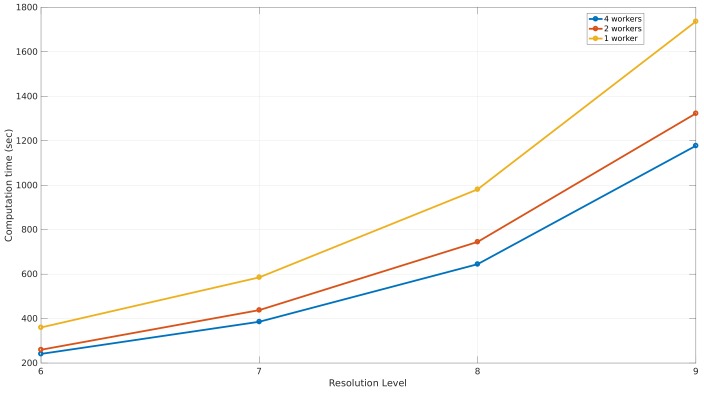
The performance of a simulation where 3200 rays are launched with different resolution levels.

This section illustrates the performance of the simulation that was used to validate this environment model by an ASUS Zenbook UX32VD, core i7-3517U in combination with 10 GB of RAM-memory. Based on the simulation parameters of the simulation that gave the best results, the computation time was measured when applying the simulation with one, two, and four processes for the different resolution levels. The results can be seen in Figure 22, where the different resolution levels are shown on the *x*-axis and the measured computation time expressed in seconds on the *y*-axis.

As a result of this analysis, applying the simulation with four workers results in the lowest computation times. The computation of a simulation at level 8 with four workers takes 644 s or 10 min and 43 s, which is 1.47 times faster than applying the simulation with one worker.

### 6.2. Office Environment 2

The second office environment consists of 453 line segments and contains 10 transmitters that were able to send DASH7 messages every 2 s at 434.56 MHz. Furthermore, the robot stopped at 22 locations and stored all messages that were received during a period of 1 min. This results in 220 individual links that are used to evaluate the ray launching propagation loss model. The results after applying GMapping in order to create a map and a transformation tree enable it to transform all receiver locations until the first robot location can be seen in Figure 14.

#### 6.2.1. Resolution

This section includes the results related to the correlation between the number of rays and the RMSE when validating the propagation model with different resolution levels of this environment model. Additionally, the two principles that were explained in Section 6.1.1 are also analyzed with this environment model. First, an evaluation is made if the average signal strength of the eight surrounding cells improves the results when a fine resolution level is applied.

**Figure 23:**
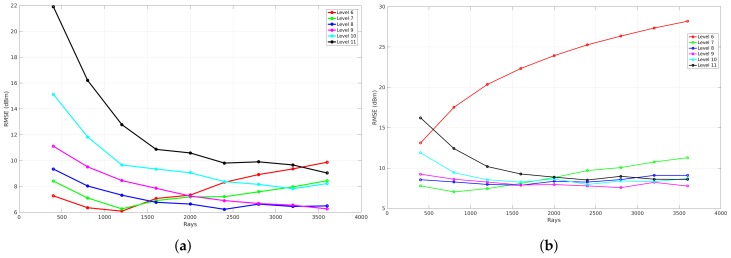
RMSE validation of the second office environment where no phase shifts are included. (**a**) zero neighbours are included; (**b**) eight neighbours are included.

Figure 23a shows the result of a benchmark where zero neighbors are included and the number resolution levels ranges from 6 to 11. Figure 23b illustrates the result when eight neighbors are included. The main difference that can be observed from both graphs is that the RMSE of resolution level 6 when eight neighbors are included increases when the number of rays increases. This means that the cell size is too large when the resolution level is equal to 6 and the influence of including the eight surrounding cells make it even worse due to the fact that the average signal strength is used. This results in an under-sampling problem. In addition to this result, the results of the validation converge when the resolution level is equal to 8 or higher.

**Figure 24:**
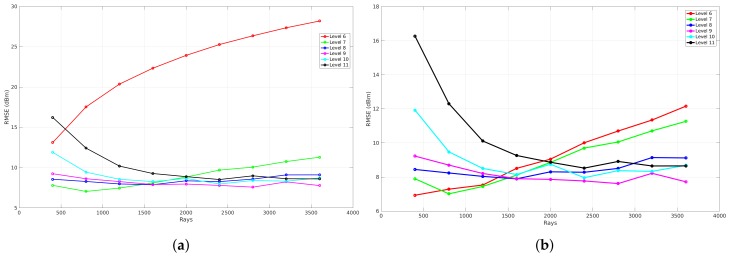
RMSE validation of the second office environment where eight neighbors are included. (**a**) no phase shifts are included; (**b**) phase shifts are included.

Furthermore, an analysis is conducted of the influence regarding whether the inclusion of the phase shifts, which happens when a ray reflects, is relevant or not when eight neighbors are included. As can be seen in Figure 24a,b, the only difference can be seen at resolution level 6. For the other resolution levels, there is no difference.

**Figure 25:**
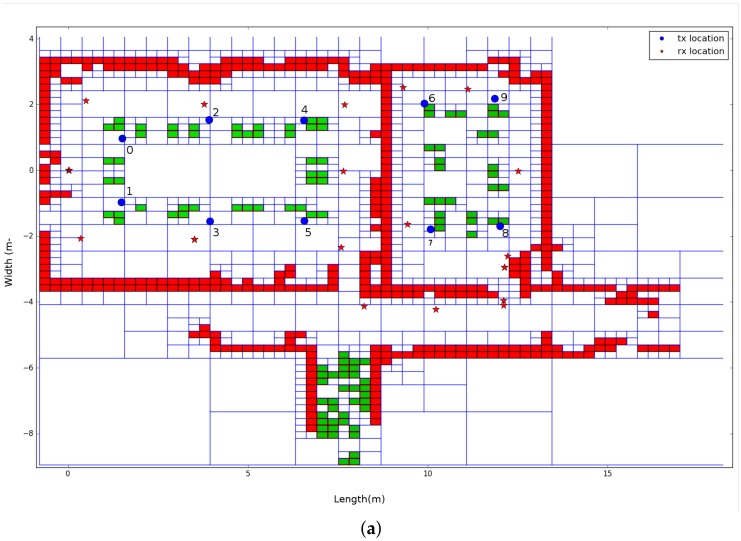

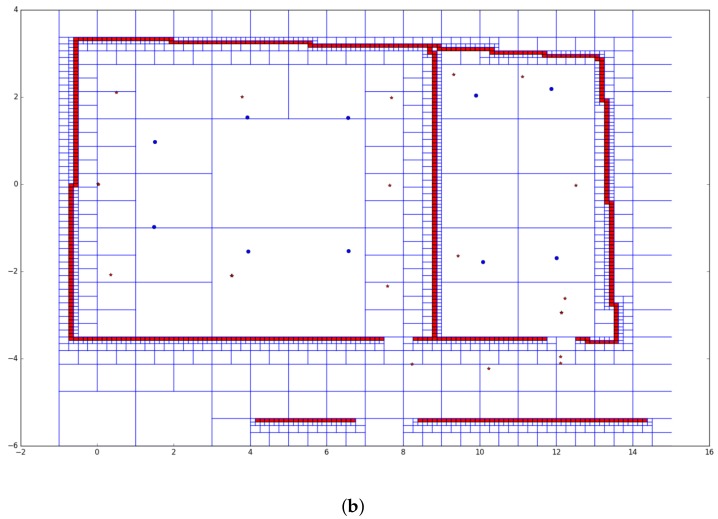
Difference between a real environment model after SLAM and a environment based on the outer boundaries of the real environment. (**a**) environment model after SLAM; (**b**) environment model of the outer boundaries.

In order to investigate the improvements of an environment model that were retrieved with a moving robot regarding an environment where there are only straight lines with the real dimensions of the environment, the same evaluation is made. Because none of the commercial software platforms use an environment model that is made from a robot, it is impossible to get a good evaluation. Furthermore, current commercial software platforms are not able to configure the resolution level of an environment model. This leads to an evaluation where we analyzed the propagation loss model with an environment model that only includes the outer boundaries of the environment. Figure 25 illustrate the differences of both environment models at resolution level 8.

**Figure 26:**
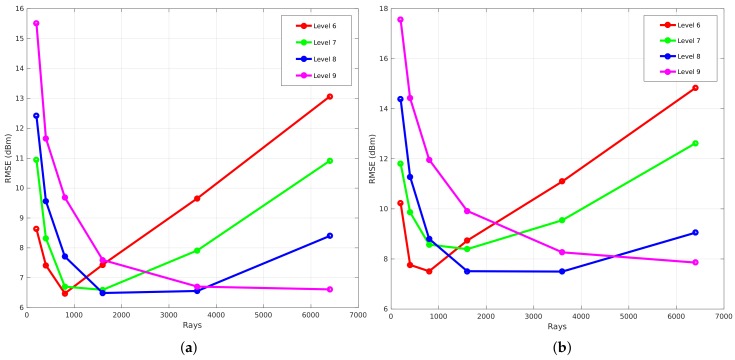
RMSE validation between a real environment model after SLAM and a environment based on the outer boundaries of the real environment. (**a**) RMSE validation of resolution levels (6, 7, 8, 9) of the environment model after SLAM; (**b**) RMSE validation of resolution levels (6, 7, 8, 9) of the environment model of the other boundaries.

To indicate the improvements, the result of a benchmark is included where no neighbors were configured and the number of resolution levels ranges from 6 to 9. As can be seen in Figure 26, the results of an environment that was captured with a robot have an average RMSE that is 3 to 4 dB more accurate than an environment where only the outer boundaries are modeled.

#### 6.2.2. Reflections

**Figure 27:**
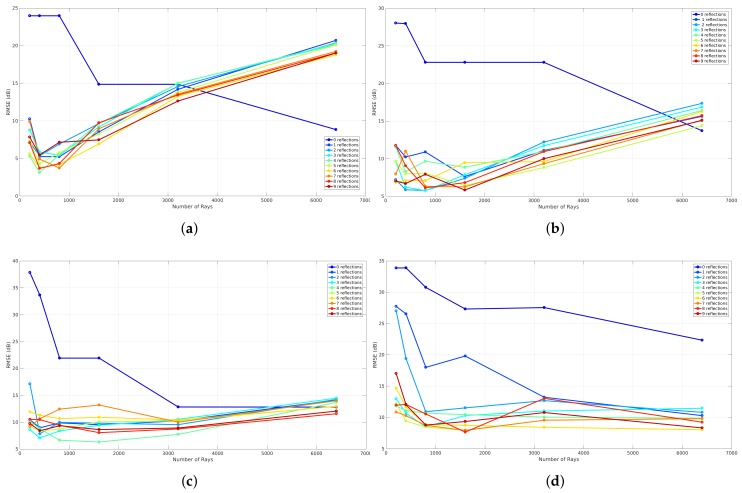
Reflection benchmark for office environment 2. (**a**) reflection benchmark at level 6; (**b**) reflection benchmark at level 7; (**c**) reflection benchmark at level 8; (**d**) reflection benchmark at level 9.

The evaluation of the reflection benchmark for this environment is also applied on four resolution levels, which are level 6 (29.7 cm ×20.3 cm), 7 (14.8 cm ×10.1 cm), 8 (7.4 cm ×5.08 cm), and 9 (3.7 cm × 2.5 cm) because every validation requires an environment model where all line segments exist of a material parameter ϵ. As a result of this environment, two values are assigned and this will be explained in the following Section 6.2.3. The results of this benchmark illustrate the global accuracy in terms of the RMSE related to the number of rays for validation where nine reflection recursion levels were applied. As can be seen in the following Figure 27, the results where the reflection recursion level was configured to zero, the RMSE decreases when the number of rays increases. Moreover, the results that were observed from the first environment in Section 6.1.2 can also be observed with this environment. This means that the global accuracy where the resolution level was 6 first decreases to a minimum where the number of rays was equal to 400 and 1600 when the resolution level was configured to 7. On the other hand, when the number of rays further increases, the RMSE gets worse for both resolution levels. Besides this behavior, in the case when resolution levels are 8 and 9, the global accuracy converges to a minimum. As a conclusion for this environment, the reflection recursion level 5 was found to be optimal for validation of the propagation loss model.

#### 6.2.3. Validation

**Figure 28:**
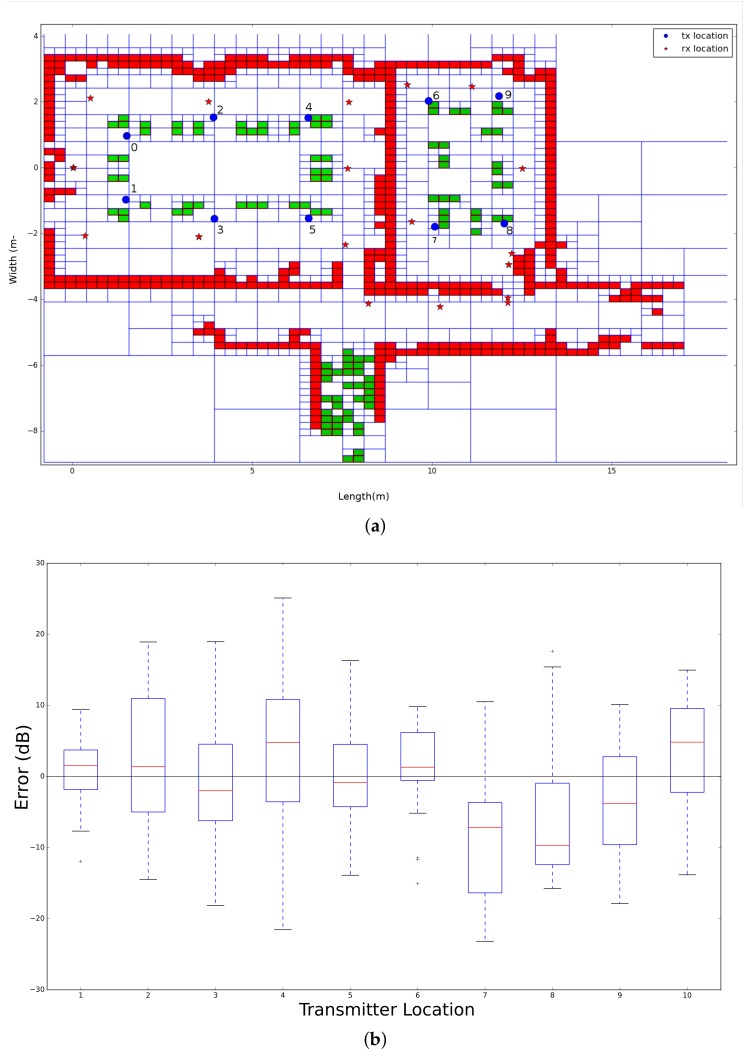
Office environment model 2. (**a**) environment model where blue dots are indicating the transmitter locations and the red stars are indicating the transmitter locations. Next, the green cells represent objects and the red cells represent a wall; (**b**) validation of each individual transmitter.

In order to validate the ray launching propagation loss model with this office environment, a Quadtree is built according to the region growing line extraction process, which is described in Section 4.1. This algorithm allows the user to assign a permittivity ϵ to each segmented cluster that was found. As a result of this, two values are used in this environment, which can be seen in Figure 28a. Due to the fact that a 2D map and monopole antennas are applied to validate the propagation loss model, all cells that are indicated by a green color get a permittivity of 1, which is the same as air. These cells were in reality table legs and did not have any influence on the received signal strength. Furthermore, all cells that are indicated by a red color represent a wall that get a permittivity of 3.

To evaluate the validation process of this office environment, Figure 28b shows the results of the individual transmitter location related to the error between the averaged measured signal strength and simulated signal strength. The results that were found optimal were based on 2700 rays, resolution level 8 (7.4 cm ×5.08 cm), and a reflection recursion level of 5. In addition to this configuration, phase shifts were included and zero surrounding cells were used.

The global accuracy of all transmitters that was analyzed for this environment can be seen in Table 3.

According to these validation results, the simulation of the transmitter at location 1 performed the best in terms of the different statistical estimators. Furthermore, a global result can be extracted by computing the average of the individual statistical estimators and can be found in Table 4.

**Figure 29:**
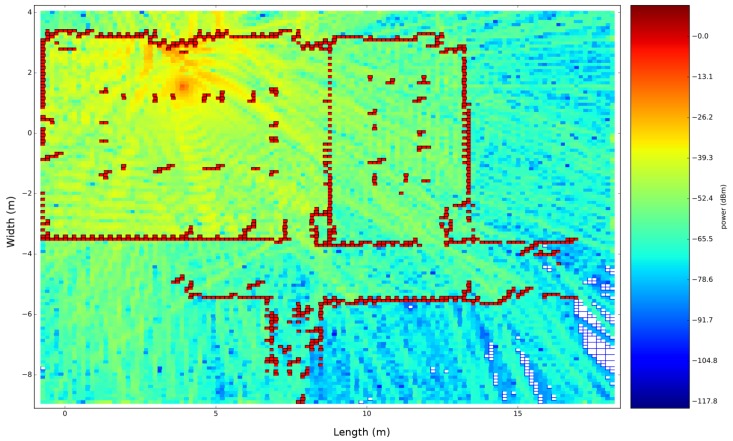
Heatmap of transmitter number two.

As a result of this evaluation, the propagation loss model performs best related to real measurements when the transmitter is located at position 1. This global RMSE of 7.69 dB is acceptable according to the different assumptions that we made. Since the RMSE is sensitive to outliers, the mean error (ME) is often used to indicate how good a radio propagation model is performing given a set of measurements. This value has to be zero in an ideal scenario. As a result of our analysis, this value is −0.81 dB, which indicates that our model is performing well. Figure 29 shows the results in the form of a heatmap, which illustrated the simulated signal strength on top of the map that was generated by a moving robot.

#### 6.2.4. Performance

**Figure 30:**
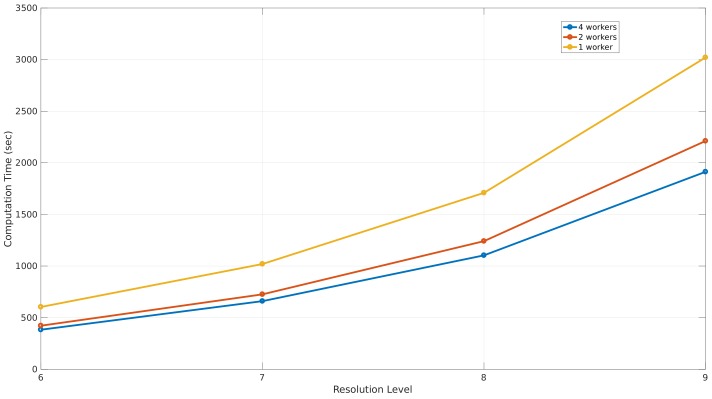
The performance of a simulation where 2700 rays are launched with different resolution levels.

The ability of applying the ray calibration process and electrical field computation in parallel improves the computation times in such a way that the speed-up when using four workers is 1.58 times faster than one worker as can be seen in Figure 30. The main difference between these performance results and those of the first office environment is that the computation time of this environment is higher than the first office environment. This difference can be explained by the fact that the number of line segments in this environment is 453 compared to 221 in the first environment, which results in more calculations to find intersections between a ray and a line segment.

The computation time of a simulation that performs best according to the simulation parameters found in Section 6.2.3 is 1103 s or 18 min and 22 s.

## 7. Conclusions

In conclusion, a realistic ray launching propagation loss model is discussed based on the implementation that contains four different parts. First, a line segment extraction algorithm is explained, which enables the classification and segmentation of a 2D-map that is created by a moving robot. Second, a specific device configuration is discussed that makes it possible to configure a ray-launching propagation simulation. Third, the core of the propagation model, which is called the ray calibration process, is discussed. The fourth part is the electrical field computation process, which uses the result of the ray calibration process as input to compute the individual electrical field values at each location that every ray visited. This results in the possibility to compute a heatmap that can be used for applications such as the optimization of localization algorithms. In addition to the implementation of the propagation loss model, this research article proposed a generic validation model that uses the result of a SLAM algorithm in combination with a set of radio measurements that were received from a transmitter at different locations. This will be used to validate and evaluate the propagation loss model by two environments. The evaluation of both environments is discussed by four perspectives, which describes first-hand the correlation between the number of rays and the resolution of the modeled environment. This evaluation is further analyzed by researching the influence of phase shifts and including the surrounding signal strengths. As a conclusion of this evaluation, every resolution level has a global accuracy in terms of RMSE that is below 8 db, which can be found when the phase shifts are included and the neighborhood is not included. Additionally, optimal results were found for both environments when the resolution level is equal to 8 or higher. Besides the correlation between the number of rays and the global accuracy, an analysis is made to evaluate the reflection recursion level regarding the number of rays and the resolution level. As a result of this evaluation, an optimal reflection recursion level of 5 is found since the accuracy is not improving when a higher level is applied. When combining these conclusions, a validation can be applied where 96 links are used for the first environment, which results in an RMSE of 9.32 dB, ME of −0.48 dB, MAE of 9.26 dB, and a precision of 5.89 dB. The results for the second environment, which are based on 220 links, after applying the validation model, are 7.69 dB for RMSE, −0.81 dB for ME, 7.64 dB for MAE, and have a precision of 5.31 dB. Finally, the performance in terms of computation time on a high performance notebook is analyzed based on the simulation configuration that gave the best results. This results in a computation time of 10 min and 43 s for the first environments and 18 min and 22 s for the second environment. Both results were computed 1.5 times faster with four workers compared to the simulation where one worker was used.

## Figures and Tables

**Table 1:** Validation results of office environment 1.

Transmitter	$E_{\mathrm{MAE}}$ (dB)	$E_{\mathrm{ME}}$ (dB)	$E_{\mathrm{RMSE}}$ (dB)	σ (dB)
1	8.17	3.99	8.20	4.12
2	9.20	1.67	9.28	7.60
3	5.38	−0.29	5.50	5.19
4	10.32	3.09	10.40	6.27
5	6.92	4.19	6.94	5.038
6	15.55	−15.55	15.56	7.13

**Table 2:** Overall results of office environment 1.

$E_{\mathrm{MAE}}$ (dB)	$E_{\mathrm{ME}}$ (dB)	$E_{\mathrm{RMSE}}$ (dB)	σ (dB)
9.26	−0.47	9.31	5.89

**Table 3:** Validation results of office environment 2.

Transmitter	$E_{\mathrm{MAE}}$ (dB)	$E_{\mathrm{ME}}$ (dB)	$E_{\mathrm{RMSE}}$ (dB)	σ (dB)
1	4.17	0.27	4.22	3.50
2	7.72	2.66	7.74	6.09
3	6.65	−1.60	6.69	5.82
4	9.77	3.23	9.79	6.84
5	5.91	0.57	6.03	4.76
6	4.99	0.49	5.09	4.48
7	10.79	−8.39	10.85	6.57
8	10.24	−5.66	10.28	4.97
9	8.63	−3.64	8.64	5.59
10	7.49	3.96	7.59	4.44

**Table 4:** Overall results of office environment 2.

$E_{\mathrm{MAE}}$ (dB)	$E_{\mathrm{ME}}$ (dB)	$E_{\mathrm{RMSE}}$ (dB)	σ (dB)
7.64	−0.81	7.69	5.31
